# Practical implication of nitroglycerin test for diagnosing heart failure in emergency department

**DOI:** 10.1002/clc.23722

**Published:** 2021-08-29

**Authors:** Shunta Hiroi, Toshihide Izumida, Teruhiko Imamura

**Affiliations:** ^1^ Second Department of Internal Medicine University of Toyama Toyama Japan; ^2^ Department of Internal Medicine Kurobe City Hospital Toyama Japan

To the Editor

We have read with great interest the article of Sekma et al., demonstrating that the impact of sublingual administration of nitroglycerin on cardiac output was useful to diagnose heart failure in patients complaining of undifferentiated dyspnea at the emergency department.^1^ We have several concerns that should improve their findings.

In their study, a decrease in cardiac output following nitroglycerin administration was greater in patients with preserved ejection fraction than those with reduced ejection fraction,^1^ but the amount seems to be comparable between those with preserved ejection fraction and those without heart failure. It might be challenging to distinguish heart failure with preserved ejection fraction from non‐heart failure diseases. Other data including plasma B‐type natriuretic peptide might eventually be required for the definitive diagnosis of heart failure with preserved ejection fraction.

In their study, cardiac output seems to decrease following nitroglycerine administration in most heart failure patients. Cardiac output would rather increase in heart failure patients on the descending part of the Starling curve when their preload was reduced. Their finding might not be applicable to those with severe congestion.

It should be practical to more clarify the optimal timing to perform nitroglycerin test. In real‐world daily practice, rapid surveillance to identify the etiology of dyspnea is required in the emergency department. Of note, transthoracic echocardiography is a powerful tool for such a purpose.^2^ The nitroglycerin test seems to take over 10 minutes. It might be challenging to perform the test before echocardiography in the fear of diagnostic delay. Furthermore, the nitroglycerin test might be contraindicated for those with right ventricular infarction, hypertrophic obstructive cardiomyopathy, and severe aortic stenosis.^3^
